# Genome-Wide Identification and Expression Pattern Analysis of SBP Gene Family in *Neolamarckia cadamba*

**DOI:** 10.3390/genes16040460

**Published:** 2025-04-17

**Authors:** Linhan Tang, Keying Li, Chuqing Cai, Wenjun Wu, Guichen Jian, Ziming Lei, Changcao Peng, Jianmei Long

**Affiliations:** Guangdong Key Laboratory for Innovative Development and Utilization of Forest Plant Germplasm, College of Forestry and Landscape Architecture, South China Agricultural University, Guangzhou 510642, China; tanglinhan0721@163.com (L.T.); crel8235@163.com (K.L.); caichuqing1224@163.com (C.C.); xh1091848031@163.com (W.W.); gustave017@163.com (G.J.); 19881774796@163.com (Z.L.); ccpeng@scau.edu.cn (C.P.)

**Keywords:** *Neolamarckia cadamba*, SBP, expression pattern, bioinformatic analysis

## Abstract

**Background**: SQUAMOSA promoter-binding protein (SBP) genes encode a group of plant-specific transcription factors that play crucial roles in plant growth, development, and stress responses. To date, *SBP* genes have been reported in a number of plant species, but the SBP gene family has not been identified in *Neolamarckia cadamba*, an important fast-growing species referred to as a ‘miracle tree’ and recognized for its potential medicinal value in Southeast Asia. **Methods**: Bioinformatics approaches were employed to conduct a comprehensive analysis of the NcSBP gene family, including investigations into physicochemical characteristics, phylogenetic relationships, gene structure, chromosomal localization, conserved motifs, *cis*-acting elements, and expression patterns. **Results**: A total of 27 *NcSBP* members were identified in the *N. cadamba* genome, encoding proteins ranging from 148 to 1038 amino acids in length, with molecular weights between 16,714.34 and 114,331.61 Da. They were classified into eight clades according to phylogenetic analysis, and unevenly distributed across 17 chromosomes, with 4 tandem gene duplication pairs and 27 fragment duplication events. In addition, *cis*-acting elements associated with hormone and light responses were most presented in the promoters of *NcSBP* genes. The transcript levels of *NcSBP* were investigated through RNA-seq and qRT-PCR, indicating distinct expression patterns across various tissues and under different hormone and stress conditions. **Conclusions**: In summary, this study comprehensively identified and characterized the SBP gene family in *N. cadamba*, providing a significant foundation for further functional investigation into *NcSBP* genes.

## 1. Introduction

The SQUAMOSA promoter-binding protein (SBP) gene family belongs to a class of plant-specific transcription factors that are widely distributed in higher plants [1]. They are characterized by an SBP domain consisting of 76 amino acid residues and contain two typical zinc finger structures and a nuclear localization signal (NLS) at the C-terminus [2]. The conserved SBP domain of SBP transcription factors has been demonstrated to be essential for binding to the palindromic GTAC core motif [3]. The SBP gene was first identified in *Antirrhinum majus* by isolating *AmSBP1* and *AmSBP2*, which could bind to the promoter of a floral meristem identity gene, SQUAMOSA [4]. The SBP genes were subsequently identified in *Arabidopsis thaliana* and *Zea mays*, and named SBP-like genes (SPL) [5,6]. To date, SBP genes have been identified across a diverse range of species, including algae, mosses [7], gymnosperms, and angiosperms [8]. However, these genes have not been found in prokaryotes, fungi, or animals [9].

Members of the SBP family are recognized for their role in regulating various aspects of plant growth and development, such as shoot and leaf development [10], flowering [11], fertility, and epidermis formation [12,13]. In *A. thaliana*, *AtSPL9* is involved in the formation of the epidermis on both the main stem and on the inflorescence, and it plays a role in regulating the vegetative-to-floral transition, as well as in anthocyanin accumulation [14]. *AtSPL2*, *AtSPL10*, and *AtSPL11* play vital roles in the regulation of leaf morphology, shoot maturation, and the promotion of trichome formation [15]. In addition, *SPL* is the target of microRNA156 (miR156), and the miR156-*SPL* module regulates a range of physiological and biochemical processes. For example, the miR156-*AtSPL3* regulatory module has been demonstrated to participate in mediating both vegetative phase transition and floral induction processes [16]. *AtSPL8* serves a dual function in the local regulation of certain developmental processes mediated by gibberellins (GAs): it is positively regulated in GA-mediated anther development, whereas it is negatively regulated in seedlings [17]. In maize, the SBP transcription factor *tsh4* is associated with bract development and the establishment of meristem boundaries [6]. Furthermore, accumulating evidence has indicated the crucial involvement of SBP genes in regulating fruit development and crop yield. More than half of rice (*Oryza sativa*) *OsSPL* is specifically expressed in young panicles [18]. Notably, the *SPL* gene *TaSPL16* from wheat (*Triticum aestivum*), which is predominantly expressed in developing panicles, has been shown to significantly enhance seed yield [19]. Additionally, *TaSPL21-6D-HapII* contributed to a remarkable 9.73% increase in 1000-grain weight [20]. In grapes, 12 *VvSBPs* from grape (*Vitis vinifera*) genes were expressed at a high level during early fruit development [21]. Particularly, the tomato (*Solanum lycopersicum*) SBP gene *LeSPL-CNR* (*Colorless Non-Ripening*) has been characterized as a key regulator of fruit ripening; the methylation-mediated epigenetic modification in its promoter region leads to the inhibition of the ripening process [22].

In addition, extensive studies have revealed that the SBP genes serve as critical regulators in modulating hormone signaling pathways and orchestrating adaptive responses to diverse abiotic stresses in multiple plant species. For example, the interaction between DELLA protein and *AtSPL9* was obstructed by GA, leading to early flowering in *A. thaliana* [23]. Functional characterization revealed that *OsSPL10* plays an important role in drought stress response via the direct transcriptional regulation of the NAC (for NAM, ATAF1/2, and CUC2) transcription factor *OsNAC2*, consequently modulating reactive oxygen species (ROS) homeostasis [24]. The overexpression of *BpSPL9* enhances the active oxygen scavenging ability of salt stress and drought stress by increasing the accumulation of superoxide dismutase (SOD) and peroxidase (POD) in transgenic lines [25]. The overexpression of an SBP gene (*VpSBP16)* from the Chinese wild grapevine *Vitis pseudoreticulata* improves tolerance to salt and drought stress during seed germination, as well as in seedlings and mature plants, by modulating the salt overly sensitive (SOS) and ROS signaling pathways in transgenic *A. thaliana* [26]. Moreover, SBP genes are important regulators in copper homeostasis. *AtSBP7* could bind to the core elements of GTAC associated with copper reactions, and the overexpression of *AtSBP7* could reduce plant toxicity in response to Cu and Cd [27,28]. The overexpression of *OsSBP9* can enhance the accumulation of Cu in rice seeds, thus improving digestibility and metabolism [29].

*N*. *cadamba* is an important timber tree in Southeast Asia; it is famously known as a ‘miracle tree’ due to its super rapid growth [30]. The swift advancement of next-generation sequencing technologies has led to the identification of SBP gene families across numerous plant species [31,32,33]. However, no systematic identification or characterization of SBPs has been conducted in *N. cadamba.* The genome sequencing of *N. cadamba* has been completed, which can provide an opportunity to identify all SBP genes in *N. cadamba* [34]. In this study, we employed comprehensive bioinformatics approaches to systematically identify and characterize the SBP gene family in *N. cadamba*, including phylogenetic classification, gene structure analysis, chromosome localization, synteny analysis, conserved motif identification, *cis*-acting element prediction, and expression profiling. These results provide a basis for the subsequent exploration of the biological function of SBP genes in *N. cadamba*.

## 2. Materials and Methods

### 2.1. Identification of NcSBP Genes in N. cadamba

To identify the potential SBP genes in *N. cadamba*, the amino acid sequences of 16 SBP genes in *A. thaliana* were obtained from the TAIR database (https://www.arabidopsis.org/, accessed on 20 May 2024) and used as query sequences to search the candidate NcSBPs via the BlastP program. Meanwhile, the hidden Markov model of the SBP gene family domain (PF03110) was downloaded from the Pfam website (https://pfam-legacy.xfam.org, accessed on 20 May 2024). The simple HMM Search program from TBtools (v2.210) was used to search all the potential SBP-containing domain protein sequences of *N. cadamba*. In these two ways, the candidate SBP proteins were obtained and submitted to the NCBI Conserved Domain Search Service (CD Search) (https://www.ncbi.nlm.nih.gov/Structure/cdd/wrpsb.cgi, accessed on 21 May 2024) to confirm their core domain sequences. The identified *NcSBP* gene was then named according to the chromosome location. The basic physicochemical properties of the NcSBP protein were analyzed with ExPASy (http://www.expasy.org/, accessed on 23 May 2024) ProtParam. The genome sequence and annotation information of *N. cadamba* was obtained from the National Center for Biotechnology Information (NCBI) database with the accession number PRJNA650253.

### 2.2. Multiple Sequence Alignment and Phylogenetic Analysis

Multiple sequence alignment of the SBP domain of the NcSBP proteins was conducted using DNAMAN software to confirm the conservation of the SBP domain. The amino acid sequences of the *A. thaliana*, *O*. *sativa*, *Populus trichocarpa,* and *N. cadamba* SBP proteins were collected for phylogenetic analysis. The accession numbers of these SBP proteins from *A. thaliana, O. sativa,* and *P. trichocarpa* are listed in Supplementary Table S1. The phylogenetic tree was constructed using the maximum likelihood (ML) method in the “One Step Build a ML Tree” program from TBtools with default parameters, with 5000 bootstrap replicates. The Interactive Tree of Life (iTOL) (https://itol.embl.de/, accessed on 26 May 2024) was used to visualize and optimize the tree subsequently. The polygenetic tree for *N. cadamba* SBP proteins was also constructed using the ML method in MEGA X with 1000 bootstrap replicates.

### 2.3. Gene Structures, Conserved Motifs, and Domain Analysis

The exon–intron structures of the *NcSBP* genes were generated using TBtools based on their genome DNA sequence and coding sequence (CDS). Multiple Expectation Maximization for Motif Elicitation (MEME) version 5.5.7 (https://meme-suite.org/meme/index.html, accessed on 28 May 2024) was used to identify the conserved motifs of NcSBP proteins, with the number of maximum motifs set to 10. The conserved domains of NcSBP proteins were searched using the NCBI’s Conserved Domain Database (CDD) (https://www.ncbi.nlm.nih.gov/Structure/cdd/wrpsb.cgi, accessed on 28 May 2024). The gene structures, conserved motifs, and domains were visualized with TBtools.

### 2.4. Chromosome Localization and Collinearity Analysis

The location of *NcSBP*s on the chromosome was examined and mapped using the plug-in program of Gene Location Visualize from GTF/GFF in TBtools software. To identify the patterns of gene duplication, synteny analyses of the SBP genes in *N. cadamba* vs. *A. thaliana* and *N. cadamba* vs. *P. trichocarpa* were conducted using the Dual Systeny Plot program of TBtools.

### 2.5. Promoter cis-Acting Element Analysis

The 2 kb upstream of the transcriptional start site (ATG) of the *NcSBP* genes was selected and considered as the gene promoter sequence. The *cis*-acting elements of the *NcSBP* promoter were predicted using the PlantCARE online website (https://bioinformatics.psb.ugent.be/webtools/plantcare/html/, accessed on 29 May 2024) and visualized with TBtools.

### 2.6. Expression Pattern of NcSBPs

The expression patterns of the *NcSBP*s were analyzed using previous transcriptome data. The different tissues, including young leaves, old leaves, bud, bark, phloem, cambium, fruit, and root from 5-year-old *N. cadamba* were sampled for RNA-seq in a previous study [35]. Three different types of vascular cells (cambium, phloem, and xylem) at three developmental stages (primary growth, secondary growth, and the transition from primary to secondary growth) were isolated via laser microdissection and used for RNA sequencing [35]. These RNA-seq data were downloaded from the NCBI under accession number SAMN15700859. The RNA-seq data for 1-aminocyclopropane-1-carboxylic acid (ACC, the precursor of ethylene) treatment were obtained from the Genome Sequence Archive (https://ngdc.cncb.ac.cn/gsa/, accessed on 2 June 2024) with submission number CRA005285 [36]. Briefly, the *N. cadamba* seedlings (height of 4–5 cm) were divided into two groups: one treated with ACC (50 μmol/L, Sigma-Aldrich, St. Louis, MO, USA) and the other with sterilized water as the control (CK). The second internode segments (counted from the apex downward) of seedlings were collected for transcriptome sequencing after ACC treatment for 6 h (6 h), 3 days (3 d), 7 days (7 d), and 14 days (14 d) [36]. For the auxin, drought, and salt stress treatments, 2-month-old seedlings of *N. cadamba* were transferred into MS liquid medium (MS basal salts (4.74 g/L), sucrose (30 g/L), adjusted to pH 5.8) supplied with 100 μmol/L indole acetic acid (IAA, Sigma-Aldrich, St. Louis, MO, USA), 10% polyethylene glycol (PEG) 6000 (Macklin Biochemical, Shanghai, China) solution, and 100 mM/L NaCl (Macklin Biochemical, Shanghai, China), respectively. The leaves were collected at 1 h, 4 h, 12 h, and 24 h after treatment. For methyl jasmonate (MeJA) (Sigma-Aldrich, St. Louis, MO, USA) treatment, the hairy root of *N. cadamba* was subjected to the MS liquid medium with 250 μmol/L MeJA and sampled after 2 h, 4 h, 8 h, 12 h, 24 h, 48 h, 72 h, and 96 h, respectively. For cold stress treatment, 3-month-old plants were transferred to a growth chamber at 4 °C, and leaves were collected after 2 h, 4 h, 8 h, 12 h, and 24 h, respectively. Three biological replicates were collected for each sample. All samples were immediately frozen in liquid nitrogen and kept at −80 °C until RNA extraction. Total RNA were extracted employing the E.Z.N.A.^®^Plant RNA Kit (Omega Bio-tek, Inc., Norcross, GA, USA) and used for subsequent RNA-seq (data not published). The expression levels of SBP genes in *N. cadamba* were quantified using fragments per kilobase of exon model per million mapped reads (FPKM). TBtools software was used to visualize the *NcSBP* expression heat map. All the FPKM values for each *NcSBP* in different tissues and under various treatments are listed in Supplementary Table S2.

Quantitative real-time PCR (qRT-PCR) was used to verify the expression patterns of the *NcSBP* genes. First-strand cDNA was synthesized via the reverse transcription of 1.0 μg total RNA using HiScript^®^ Ⅲ RT SuperMix for qPCR Kit (R323, Vazyme Biotech, Nanjing, China). qRT-PCR was performed utilizing SYBR Green mix (Vazyme Biotech, Nanjing, China) on a Roche LightCycler^®^ 480 instrument. The cycling parameters were as follows: 95 °C for 30 s, 40 cycles at 95 °C for 10 s, and 60 °C for 30 s. Melt-curve analyses were performed using the following program: 95 °C for 15 s, 60 °C for 60 s, and 95 °C for 15 s. Three biological replicates and four technical replicates were used for each sample. The relative expression of genes was analyzed using the 2^−ΔΔct^ method, with the reference gene of *NcUPL* (ubiquitin–protein ligase) [37]. Significance was determined by multiple comparisons using ANOVA (*p* < 0.05). The gene-specific primers used in qRT-PCR are listed in Supplementary Table S3.

## 3. Results

### 3.1. Identification and Phylogenetic Analysis of NcSBP Genes

In this study, a total of 27 SBP gene family members were identified in *N. cadamba* and named as *NcSBP1*-*NcSBP27,* according to their position on chromosomes. The analysis of the gene characteristics of the NcSBP proteins showed that the lengths of all the identified *NcSBPs* ranged from 148 to 1038 amino acids. Of these, *NcSBP19* was the largest protein and *NcSBP9* was the smallest protein. The molecular weight (MW) of the *NcSBPs* varied from 16,714.34 to 114,331.61 Da, with the isoelectric point (pI) ranging from 5.64 to 9.76. Interestingly, the pI of 20 *NcSBPs* was more than 7, while only 7 *NcSBPs* had a value less than 7. The instability index of the *NcSBPs* ranged from 35.66 to 97.12, and the aliphatic index was between 40.0 and 85.13. The grand average of hydropathy of the *NcSBPs* was between −1.400 and −0.399, indicating that they were all hydrophilic proteins (Table 1).

**Figure 1:**
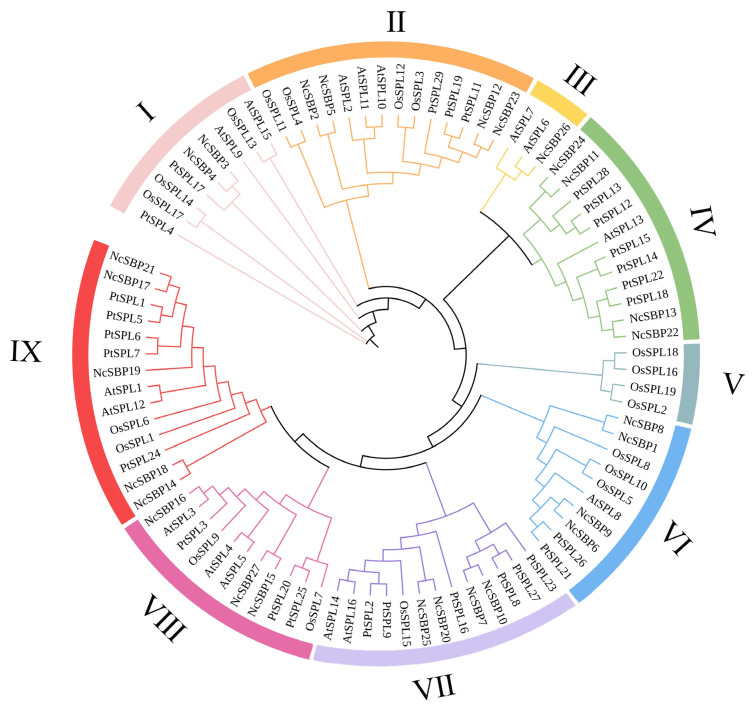
Phylogenetic tree of SBP proteins from *N. cadamba*, *O. sativa*, *P. trichocarpa*, and *A. thaliana*. The SBP protein sequences of 27 *NcSBPs*, 16 *AtSBPs*, 19 *OsSBPs,* and 29 *PtSBPs* were used to construct the phylogenetic tree using the maximum likelihood (ML) method with 5000 bootstrap replicates. The subfamilies of SBP proteins, clade I~IX, were marked with different-colored arcs. Nc: *N. cadamba*; Os: *O. sativa*; Pt: *P. trichocarpa*; At: *A. thaliana*.

To verify the conserved SBP domain in each *NcSBP* protein, multiple sequence alignment was performed using DNAMAN software. The results showed that the SBP domains in all NcSBP proteins exhibited conserved structural features, including two characteristic zinc-finger motifs (C3H and C2HC) and a bipartite nuclear localization signal (NLS). However, there was an exception that no NLS was found in *NcSBP6* (Figure S1). To further investigate the phylogenetic relationships among the SBP proteins, an ML phylogenetic tree was generated using the SBP proteins from *N. cadamba*, *A. thaliana*, *O. sativa,* and *P. trichocarpa.* The results displayed that the SBP proteins were divided into nine clades (clade Ⅰ to clade Ⅸ) (Figure 1), according to the classification established in a previous study on *A. thaliana* and rice [38], suggesting that there is evolutionary conservation between these four species. The number of SBP members varied between all nine clades. Clades Ⅱ and Ⅸ had the largest numbers of SBP proteins, with 14 members in each clade, respectively. NcSBP proteins were distributed in all clades except clade Ⅴ, which was specific to the monocot plant. There was a differential distribution of *NcSBP* members across various clades, with the highest representation observed in clade IX (five members) and the lowest in clade III (one member).

### 3.2. Conserved Motif and Gene Structure Analysis of NcSBPs

**Figure 2:**
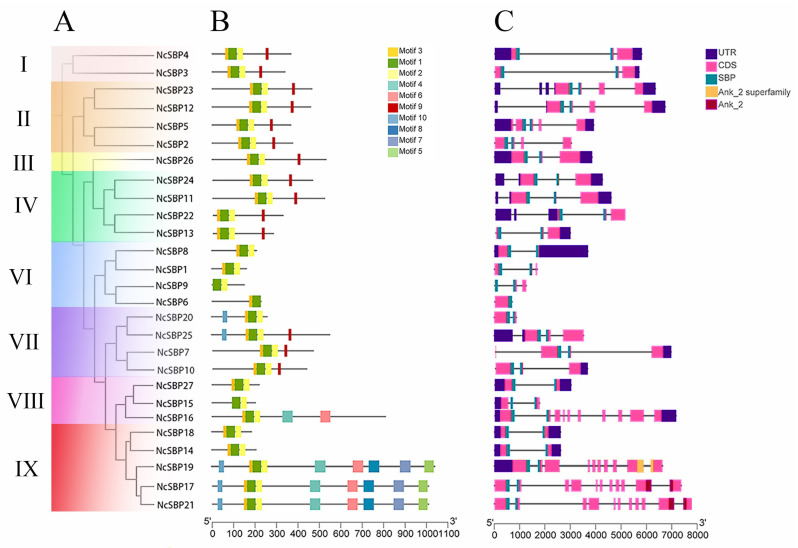
Polygenetic relationships, conversed motifs, and gene structure of *NcSBP* genes. (**A**) The polygenetic tree was constructed based on the 27 NcSBP protein sequences using the maximum likelihood (ML) method with 1000 bootstrap replicates. The subfamilies of NcSBP, clade I to clade IX except clade V, were shown in different colors as the same of Figure 1. (**B**) Conversed motif composition of NcSBP proteins. Ten motifs (motif 1~motif 10) are represented with different colored boxes. (**C**) Exon–intron structure of *NcSBP* genes. Exons and untranslated regions (UTRs) are shown in purple and pink boxes, and introns are displayed with black lines. SBP-conserved domain is shown in a light green box, and other colors indicate different conserved domains found in CDD.

An unrooted phylogenetic tree was constructed to align the amino acids of 27 *NcSBPs* from *N. cadamba* (Figure 2A). The conserved motifs within the NcSBP protein sequences were analyzed using the MEME tool. As shown in Figure 2B, motifs 1, 2, and 3 were present in all members of the *NcSBP* family, indicating their potential significance in the biological function of *NcSBP*. Furthermore, the types and distributions of motifs among the different NcSBP proteins within the same clade exhibited high similarity. For instance, motifs 1, 2, 3, and 9 were consistently arranged in clade I, II, III, and Ⅳ, suggesting a high degree of sequence similarity among these clade members. Notably, motif 9 was identified in all members of clades I, II, III, Ⅳ, and Ⅶ, with the exception of *NcSBP20*. In contrast, motifs 4, 5, 6, 7, and 8 were predominantly found in *NcSBP17*, *NcSBP19*, and *NcSBP21*, potentially contributing to the functional specificity of these *NcSBP* transcription factors.

Additionally, the exon–intron distribution patterns of the *NcSBP* genes were investigated by comparing their coding sequences (CDS) with genomic sequences. As shown in Figure 2C, we found variability in the number of exons across different clades. For example, members in clade I (*NcSBP3* and *NcSBP4*) contained 3 exons, whereas three members in clade Ⅱ (*NcSBP2*, *NcSBP5*, *NcSBP12*, and *NcSBP23*) exhibited 4-7 exons. The *NcSBP* genes within some clades shared similar exon and intron structures, such as the presence of two introns in clade I. However, certain *NcSBP* genes displayed structural deviations from their clade counterparts. For instance, *NcSBP14* and *NcSBP18* in clade Ⅸ had significantly fewer exons compared to other genes in the same clade. Moreover, with the exception of *NcSBP1/6/9/20*, most *NcSBP* genes contained at least one non-coding region (UTR). Interestingly, all members of the *NcSBP* gene family harbored conserved SBP domains located on two exons, which were invariably separated by an intron (Figure 2C). In addition, the SBP domains of all the *NcSBP* genes, with the exception of *NcSBP6*, *NcSBP22,* and *NcSBP23*, were distributed in the first and second exons, or the second and the third exons.

### 3.3. Chromosome Localization and Collinearity Analysis of NcSBPs

**Figure 3:**
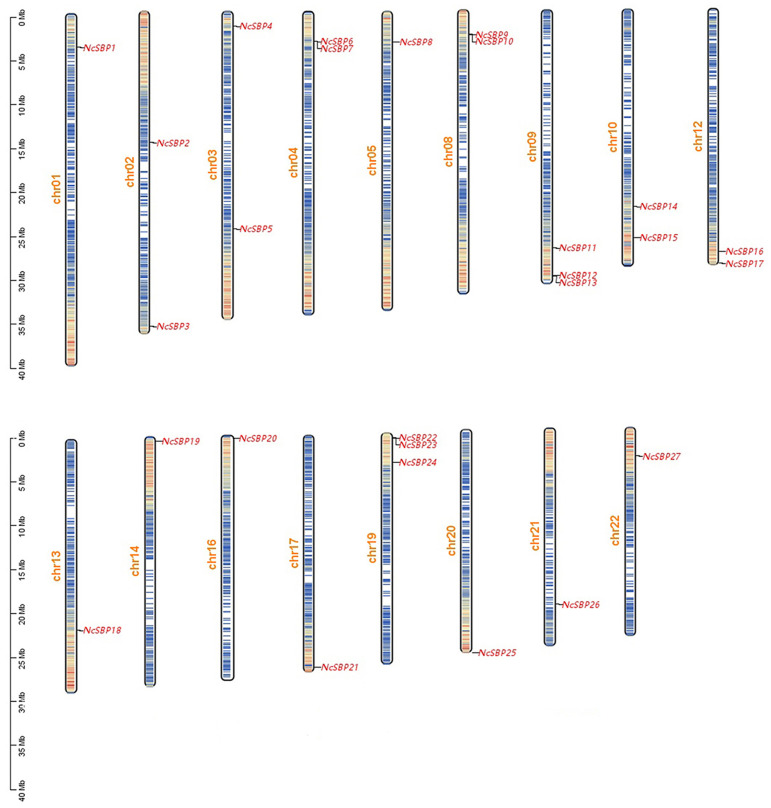
Chromosome location of *NcSBP* genes. The scale bar on the left indicates the chromosome length. Gene densities are drawn based on the annotation data of the *N. cadamba* genome, with red representing high density and blue representing low density. Red font size markers are gene names, and chr indicates chromosome.

The distribution of *NcSBP* genes across the chromosomes was predicted using the TBtools software, based on the available gene annotation information of the *N. cadamba* genome. The results showed that the 27 *NcSBP* genes were unevenly distributed on 17 chromosomes, with 1-3 *NcSBP* genes in each chromosome (Figure 3). To obtain insight into the expansion of the *NcSBP* family, we performed gene duplication analysis. In general, a gene cluster is defined as a region no longer than 20 kb and containing two or more genes from the same family. Accordingly, four *NcSBP* gene clusters (*NcSBP6*/*NcSBP7*, *NcSBP9*/*NcSBP10*, *NcSBP12*/*NcSBP13*, and *NcSBP22*/*NcSBP23*) were characterized as tandem repeat gene pairs, located on chromosomes chr04, chr08, chr09, and chr19.

**Figure 4:**
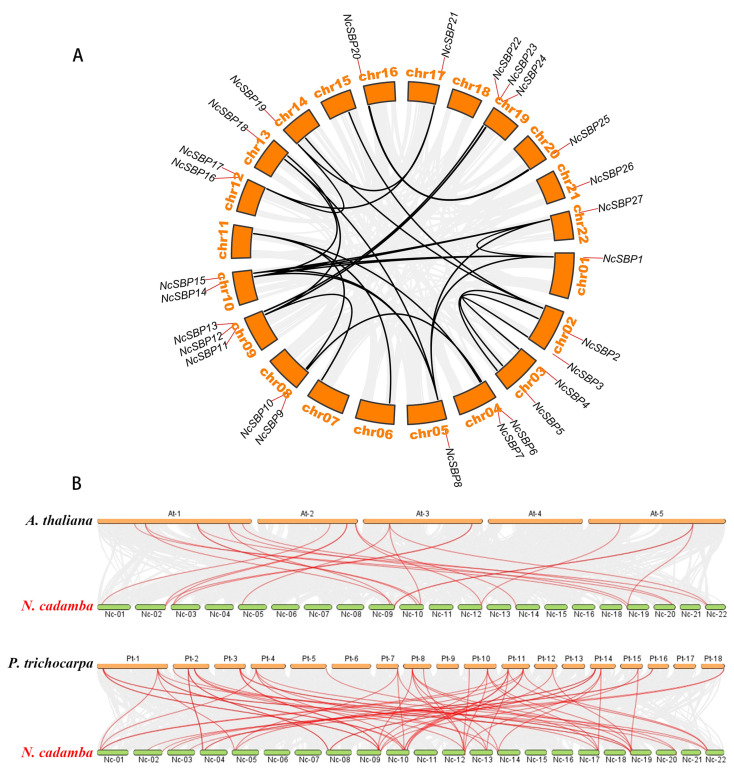
Synteny analysis of SBP genes within *N. cadamba* (**A**) and between *N. cadamba* and two representative species of *A. thaliana* and *P. trichocarpa* (**B**) Gray lines in the background represent the synteny blocks in the genomes, and black or red lines indicate duplication *SBP* gene pairs.

In addition to tandem duplication, fragment duplication events within the *NcSBP* gene family were also conducted. The intraspecific collinearity analysis identified 27 collinear pairs among the *NcSBP* gene family, encompassing 26 *SBP* genes. Notably, no collinear modules were observed on chromosomes 18 and 21 (Figure 4A). Each pair of collinear genes was situated on different chromosomes and was associated with fragment replication events. Taken together, the analysis of gene duplication events suggests that fragment replication serves as the main driving force behind the expansion of the SBP gene family.

To further elucidate the phylogenetic mechanisms of the *N. cadamba* SBP family, comparative syntenic maps were constructed, integrating *N. cadamba* with two representative species of *A. thaliana* and *P. trichocarpa* (Figure 4B). The results revealed 22 SBP orthologous gene pairs between *N. cadamba* and *A. thaliana*. They were identified between 11 chromosomes of *N. cadamba* and 4 chromosomes of *A. thaliana*. Notably, the collinearity blocks were predominantly concentrated in chromosome At-1 of *A. thaliana*. Additionally, 55 collinear gene pairs were identified between *N. cadamba* and *P. trichocarpa*. Many collinearity blocks were observed between chr15 of *N. cadamba* and chromosome Pt-14 of *P. trichocarpa*. However, no synteny blocks were found in *N. cadamba* chromosomes Nc-06, 07, 11, 15, 16, or 21, or in *P. trichocarpa* chromosomes Pt-6, 9, 13, or 17. These findings suggest that *NcSBP* genes exhibit a closer phylogenetic relationship with the SBP genes of *P. trichocarpa* compared to those of *A. thaliana*. Furthermore, certain *NcSBP* genes, such as *NcSBP27*, share multiple orthologous gene pairs with both *P. trichocarpa* and *A. thaliana.* Conversely, some genes display collinearity predominantly with one species. For instance, *NcSBP8* on chr05 of *N. cadamba* shares three orthologous gene pairs with *P. trichocarpa* but none with *A. thaliana*, suggesting its potential role in the growth and development of woody plants specifically.

### 3.4. cis-Acting Element Analysis of NcSBP Promoters

**Figure 5:**
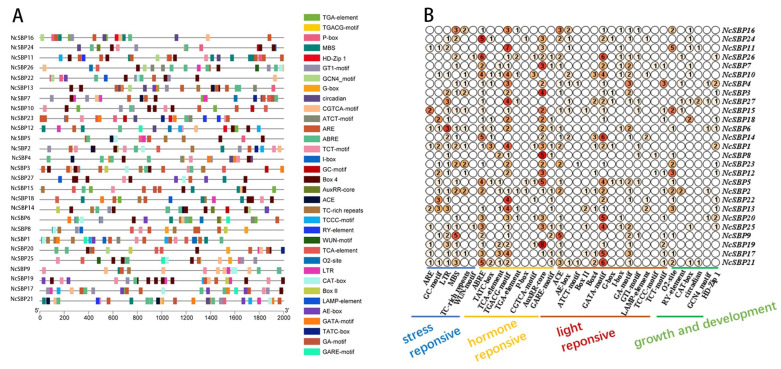
*Cis*-acting element analysis of the promoter region of *NcSBP* genes. (**A**) The distribution of *cis*-acting elements in *NcSBP* promoters. Blocks with different colors represent various types of *cis*-acting elements. (**B**) The number of *cis*-acting elements related to stress response, hormone response, light response, and growth and development in *NcSBP* promoters.

To enhance our understanding of transcriptional regulation and the possible roles of *NcSBPs* in *N. cadamba*, we predicted the *cis*-acting elements present in the promoters of *NcSBP* genes using Plant CARE (Figure 5A). The *cis*-acting elements identified in the promoter regions of *NcSBP* genes were categorized into four groups, including stress response elements, hormone response elements, light response elements, and plant growth and development response elements. Among these, hormone response elements and light response elements were the most abundant. Specifically, the promoters of *NcSBP1*, *NcSBP5*, *NcSBP10*, *NcSBP17*, *NcSBP19*, *NcSBP20*, and *NcSBP26* contained 12 or more hormone response *cis*-acting elements, suggesting their potential significance in hormone-mediated regulatory processes. Within the hormone response elements, ABRE (abscisic acid response element), TGACG motif (MeJA response element), and AuxRR core (auxin response element) were particularly prevalent. In terms of light response elements, the GATA motif was notably abundant, with *NcSBP14*, *NcSBP21*, and *NcSBP26* each containing six of such elements. Additionally, stress-related elements, including the drought-responsive MYB binding site (MBS) and the low-temperature response element (LTR), were widely distributed, appearing in over 70% of the *NcSBP* promoter regions (Figure 5B). These findings highlight the diverse regulatory roles of *NcSBP* genes in responding to environmental stresses, hormonal signals, and light conditions.

### 3.5. Expression Patterns of NcSBP Genes in Various Tissues

**Figure 6:**
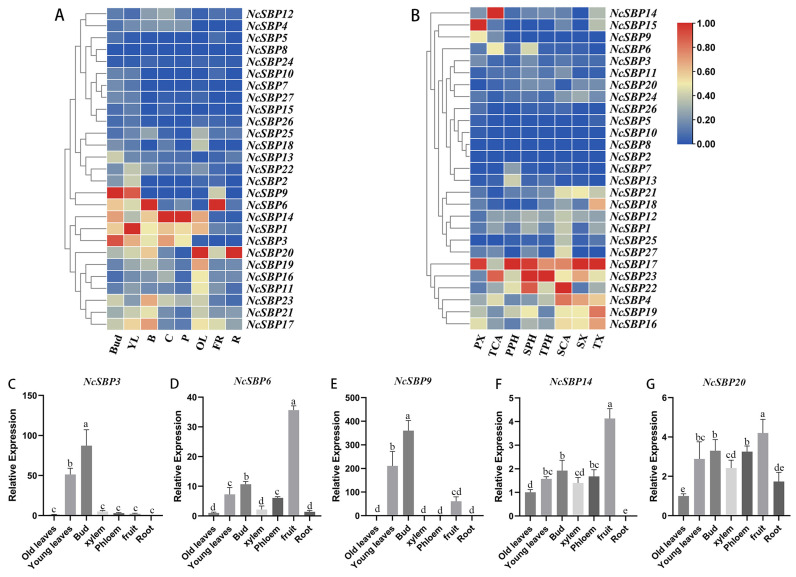
Expression patterns of *NcSBP*s in different tissues and vascular cells at three developmental stages. Expression of *NcSBP* genes in various tissues. (**A**) YL, young leaves; B, bark; C, cambium; OL, old leaves; FR, fruit; R, root. (**B**) *NcSBP*s transcript levels in cambium, phloem, and xylem cells at different developmental stages. PX, primary xylem cells; TX, xylem cells at the transitional stage from primary to secondary growth; SX, secondary xylem cells; TCA, cambium cells at the transitional stage from primary to secondary growth; SCA, secondary cambium cells; PPH, primary phloem cells; TPH, phloem cells at the transitional stage from primary to secondary growth; SPH, secondary phloem cells. Heatmaps were generated using TBtools, utilizing transformed log2 (FPKM+1) values, and cluster analysis was conducted on the gene expression levels by row. The color bar indicated gene expression, with red representing high expression and blue representing low expression. (**C**–**G**) Gene expression analysis of 5 selected *NcSBP* genes in eight tissues of *N. cadamba* by qTR-PCR. *NcUPL* was used as the reference gene, and transcript levels in old leaves were set as the calibrator (assigned a value of 1). Relative expression in other tissues was then determined accordingly. Error bars represent standard deviations of mean value from three biological replicates. Different letters indicate statistically significant differences between groups based on ANOVA (*p* < 0.05).

To investigate the possible role of *NcSBP* in the development of various tissues and organs, we analyzed the expression patterns of 27 *NcSBP* genes across different tissues, including bud, bark, young leaves, old leaves, root, young fruit, cambium, and phloem, utilizing previously acquired transcriptome data (Figure 6). The results showed that most *NcSBP* genes were expressed in diverse tissues. Notably, *NcSBP3* and *NcSBP9* exhibited high expression levels in buds, whereas *NcSBP1* demonstrated the highest expression in young leaves. *NcSBP6* was predominantly expressed in bark and young fruit, but *NcSBP14* showed elevated expression in cambium and phloem, while *NcSBP20* was specifically expressed in old leaves and roots (Figure 6A). To examine the expression profiles of *NcSBP* in various developmental vascular tissues, we employed laser microdissection to isolate cambium, phloem, and xylem cells at three distinct stages: primary growth, secondary growth, and the transitional stage from primary to secondary growth [39]. Following this, RNA sequencing was performed. The expression profiling analysis revealed distinct spatial–temporal expression patterns of *NcSBP* genes during vascular development in *N. cadamba*. Obviously, *NcSBP17* exhibited consistently high expression levels across the three developmental stages in all vascular tissues (cambium, phloem, and xylem), except in cambium cells at the transition stage, suggesting its significant role in vascular tissue differentiation and development in *N. cadamba*. Additionally, *NcSBP23* had high expression during the primary growth and transition stage of phloem development, and *NcSBP14* was specifically highly expressed during the transition stage of vascular cambium growth. Furthermore, *NcSBP15* showed high expression in the primary xylem, while *NcSBP22* was predominantly expressed in the phloem and cambium during secondary growth. These differential expression patterns strongly suggest that *NcSBP* genes functionally specialized in regulating specific stages of vascular tissue development and differentiation processes in *N. cadamba*.

To further validate the expression profiles of *NcSBP*s across various tissue types, qRT-PCR was employed to assess the expression levels of five *NcSBP*s (*NcSBP3*, *NcSBP6*, *NcSBP9*, *NcSBP14*, and *NcSBP20*) that exhibited high expression in different tissues (Figure 6C–G). Consistent with the transcriptome data, the qRT-PCR analysis revealed that *NcSBP3* and *NcSBP9* were most highly expressed in buds and young leaves, while *NcSBP6* showed strong expression in fruits (Figure 6C–E). However, discrepancies were found in the expression patterns of *NcSBP14* and *NcSBP20* compared to the transcriptome data. Specifically, *NcSBP14* was predominantly expressed in fruits (Figure 6F), whereas the transcriptome analysis indicated high expression in the phloem and cambium (Figure 6A). Additionally, *NcSBP20* demonstrated high expression in various tissues, excluding old leaves and roots (Figure 6G). It is hypothesized that the differences in expression patterns may be attributed to variations in the sample sources utilized for transcriptome analysis and qRT-PCR.

### 3.6. Expression Analysis of NcSBPs in Response to Hormones and Abiotic Stress Treatment

**Figure 7:**
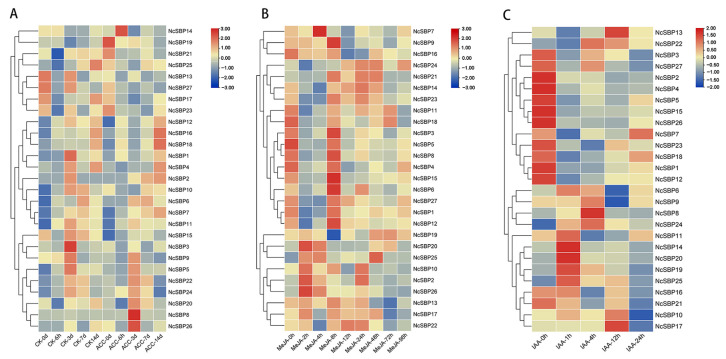
Expression pattern of *NcSBPs* at different times under various hormone treatments. (**A**) Expression levels of *NcSBPs* under ACC (1-aminocyclopropane-1-carboxylic acid, precursor of ethylene, 50 μmol/L) treatment and the sterilized water treatment (CK). (**B**) Expression levels of *NcSBPs* under MeJA (250 μmol/L) treatment. (**C**) Expression analysis of *NcSBPs* under IAA (100 μmol/L) treatment. The heatmaps were generated using TBtools based on the transcriptome data, with transformed data of log_2_(FPKM+1) values. Gene expression clustering was performed row-wise. The color bar indicated gene expression level, with red representing high expression and blue representing low expression. d: day, h: hour.

To investigate whether the *NcSBP* genes were response to different hormones, expression patterns of all the identified *NSBPs* were analyzed using the RNA-seq data. The results demonstrated that ACC treatment significantly up-regulated the expression levels of *NcSBP14* after 1 day of treatment compared to the control group. Notably, *NcSBP8* and *NcSBP26* exhibited the most pronounced up-regulation on day 3 post-treatment (Figure 7A). As for MeJA treatment, the expression of *NcSBP2*, *NcSBP20*, *NcSBP10,* and *NcSBP26* was up-regulated at 2 h after treatment initiation. The expression of *NcSBP7* peaked at 4 h, while NcSBP6/9/15 reached their highest levels under MeJA treatment at 8 h. Conversely, *NcSBP13* and *NcSBP17* showed down-regulated expression after 72 h and 96 h of treatment, respectively (Figure 7B). Under IAA treatment, the expression levels of *NcSBP1*/*2*/*4*/*5*/*12*/*15*/*23*/*25* gradually decreased with prolonged treatment time. In contrast, *NcSBP8* expression was induced and peaked at 4 h, while *NcSBP11/14/19/20/25* and *NcSBP13/17* reached their highest expression levels at 1 h and 12 h, respectively (Figure 7C). These findings indicate that the expression patterns of *NcSBP* genes varied under ACC, MeJA, and IAA treatments, suggesting that *NcSBPs* may play distinct roles in different hormone response pathways.

**Figure 8:**
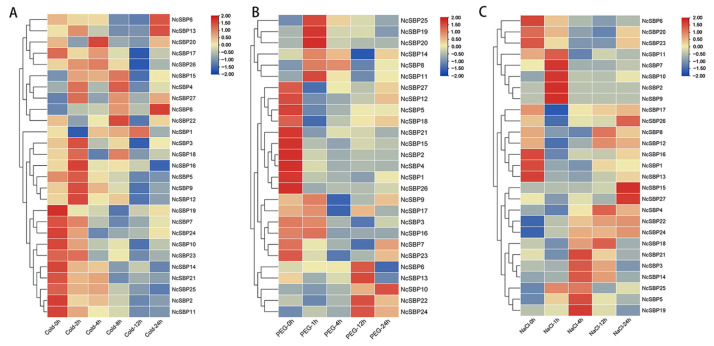
Expression pattern of *NcSBPs* at different times under cold (**A**), drought (**B**), and salt (**C**) stress. The seedlings of *N. cadamba* were treated with low temperature (4 °C), 10% PEG6000, and NaCl (100 mM/L), respectively. The heatmaps were generated using TBtools based on the transcriptome data, with transformed data of log2(FPKM+1) values. Gene expression clustering was performed row-wise. The color bar indicates gene expression, with red representing high expression and blue representing low expression. h: hour.

To elucidate the response mechanisms of *NcSBP* genes to abiotic stress, we analyzed transcriptome data under various stress treatments, including low temperature (4 °C), drought (stimulated by PEG), and salt (NaCl) (Figure 8). Under low-temperature stress, the expression levels of *NcSBP2/7/10/11/14/19/21/23/24/25* decreased gradually with prolonged treatment. In contrast, *NcSBP3/5/9/12/16* reached the highest transcript levels at 2 h post-treatment before declining. Notably, *NcSBP6*, *NcSBP8*, and *NcSBP13* exhibited delayed responses, reaching their highest expression levels after 24 h of treatment, suggesting a slower adaptation to low-temperature stress. For PEG treatment, the expression levels of *NcSBP1/2/4/5/12/15/18/21/26/27* declined over time. Conversely, *NcSBP11*, *NcSBP19*, *NcSBP20*, and *NcSBP25* peaked at 2 h before decreasing. Meanwhile, *NcSBP6/13/22/24* and *NcSBP10* reached their highest expression levels at 12 h and 24 h, respectively, with low expression at other time points. Under NaCl conditions, the expression levels of *NcSBP1/6/13/16/20/23* were significantly down-regulated. In contrast, *NcSBP2/7/9/10/11* exhibited an initial increase at 1 h, followed by a gradual decline. *NcSBP3/5/14/19/21/25* had the highest expression at 4 h, while *NcSBP15* and *NcSBP27* showed elevated expression only at 24 h, remaining low at other time points. Taken together, these results highlight the diverse and dynamic expression patterns of *NcSBP* genes under different abiotic stress conditions, suggesting their involvement in distinct stress response mechanisms.

**Figure 9:**
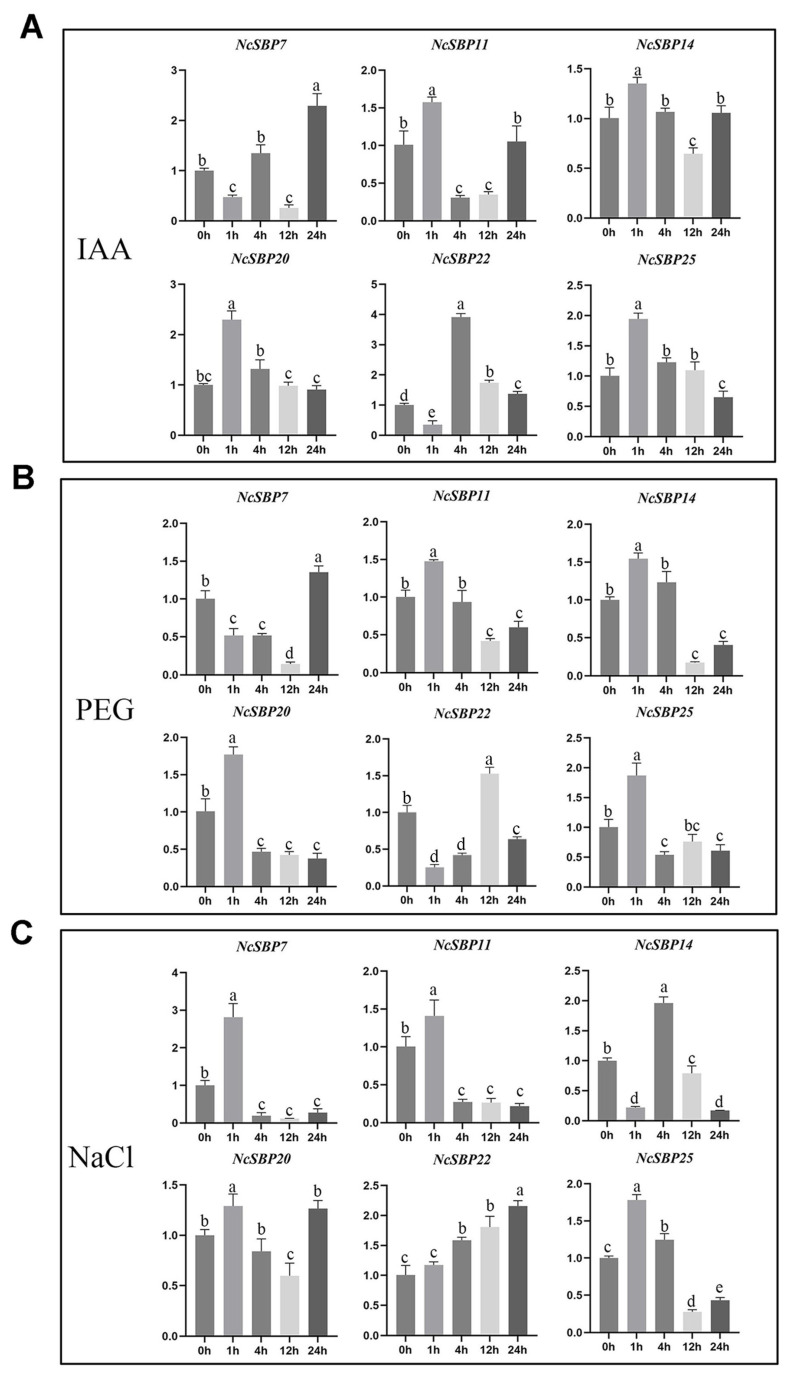
Analysis of 6 selected *NcSBPs* at different times under IAA (**A**), PEG (**B**), and NaCl (**C**) treatments by qRT-PCR. *NcUPL* served as the reference gene, with transcript levels before treatment (0 h) normalized to 1 for relative quantification in other treatments. Error bars represent standard deviations of mean value from three biological replicates. Groups marked with different letters differ significantly (ANOVA, *p* < 0.05).

To confirm the expression patterns of *NcSBPs* in response to hormone and abiotic stresses, qRT-PCR was employed to assess the expression levels of six selected *NcSBP*s (*NcSBP7*, *NcSBP11*, *NcSBP14*, *NcSBP20*, *NcSBP22*, and *NcSBP25*) under IAA, PEG6000, and NaCl treatment (Figure 9). The results showed that *NcSBP11*, *NcSBP14*, *NcSBP20*, and *NcSBP25* reached the highest expression level after 1 h under IAA and PEG treatment (Figure 9A,B), indicating a rapid response to these two treatments. On the contrary, *NcSBP7* exhibited delayed responses, reaching their highest expression levels at 24 h post-treatment. Notably, *NcSBP22* had the highest expression at 4 h under IAA treatment, whereas it exhibited the highest expression at 12 h under PEG treatment. Under the NaCl condition, *NcSBP7*, *NcSBP11*, *NcSBP20,* and *NcSBP25* reached their highest expression quantity at 1 h post-treatment (Figure 9C), indicating a rapid response to NaCl. The difference is that *NcSBP7* and *NcSBP11* remained low at other time points, while *NcSBP20* and *NcSBP25* had low expression only at 12 h. It is worth noting that *NcSBP14* reached the highest expression level at 4 h, while *NcSBP22* increased gradually with prolonged treatment. All together, these results suggest that the expression patterns of these *NcSBPs* detected by qRT-PCR were consistent with the transcriptome data.

## 4. Discussion

The SBP family is a significant transcription factor family exclusive to plants, recognized for their role in regulating flower and fruit development, as well as various other essential physiological processes. With the advancement of next-generation sequencing technologies, SBP gene families have been widely identified in numerous species. However, there is no research on SBP genes in *N. cadamba,* an important timber tree with high medicinal value in subtropical Asian regions. In the present study, we systematically identified and characterized the SBP gene family in *N. cadamba* and performed a comprehensive analysis with regard to phylogenetic relationships, protein properties, gene structure, chromosome localization, collinearity, *cis*-acting elements in promoters, expression patterns in different tissues, and responses to various hormone and abiotic stresses.

In general, the SBP gene family is relatively small in terms of transcription factors in plants, with the majority comprising fewer than 40 members. For instance, there are 16 members in *A. thaliana* [38], 19 members in rice [38], 15 members in sweet orange (*Citrus sinensis*) [40], and 32 members in blueberry (*Vaccinium uliginosum* L.) [41]. In our study, a total of 27 SBP genes were identified, which was the same as apple (*Malus × domestica Borkh*) [8]. The number of SBP genes differed among the various plant species; however, this variation did not correspond proportionally with alterations in genome size [42]. The tea plant (*Camellia sinensis*) genome size (3.14 Gb and 3.02 Gb) was much greater than *N. cadamba* (744.5 Mb), but only 20 SBP members were identified in tea plant. The variation in the number of SBP genes across different plant species may be due to gene duplication or the prolonged expansion of certain LTR retrotransposon families [43,44]. Generally, gene duplication events, including segmental and tandem duplications, are significant contributors to the emergence of new genes and the expansion of gene families, facilitating the adaptation of organisms to diverse and complex environments. To date, tandem duplication and segmental duplication have been extensively characterized within the SBP gene family. In our study, we identified four gene clusters *(NcSBP6/NcSBP7*, *NcSBP9/NcSBP10*, *NcSBP12/NcSBP13*, and *NcSBP22/NcSBP23*), which were classified as tandem duplicate pairs in *N. cadamba*. Additionally, two tandem duplicate pairs of 16 *SBP* genes in *A. thaliana* were located in the segmental repeat region [38]. In perennial plants, 11 pairs of 29 SBP genes in *P. trichocarpa* arose from intrachromosomal duplication [44], while 27 inter-chromosomal segmental duplication events were identified among 28 *EjSBP* genes in loquat (*Eriobotrya japonica*) [45]. In our study, we identified 27 pairs of genes situated within duplicated genomic regions in *N. cadamba* (Figure 3). These findings suggest that the duplication in SBP gene family members is widespread and relatively conserved across the plant.

Gene structure analysis revealed that *NcSBP* genes exhibited a range of 1 to 11 exons, likely resulting from the evolutionary processes of intron and exon insertion and deletion within *NcSBP*s. However, the majority of genes consisted of three to four exons, suggesting that *NcSBP*s exhibit a relatively conserved structure. According to the phylogenetic tree, 27 *NcSBPs* were categorized into eight distinct subfamilies, which was similar to the phylogenetic structure observed in *AtSPLs* [38]. Members of *A. thaliana* and *N. cadamba* were found across various subfamilies, with *NcSBPs* within the same subfamily exhibiting comparable motifs and structural features (Figure 2). This suggests that these genes may have originated from a common ancestor and might serve analogous roles in plant growth and development. Furthermore, the majority of *NcSBPs* exhibited a closer clustering with SBP genes from *A. thaliana* and *P. trichocarpa*, rather than those from rice (Figure 1). This finding is consistent with the knowledge that *A. thaliana* and *P. trichocarpa* are eudicots, which separated more recently from a common ancestor than the lineage that gave rise to monocots. Comparative genomic analysis provides a powerful approach for extrapolating genomic insights gained from one taxon that has been extensively investigated in terms of its genome structure, biological function, and evolutionary dynamics to less-studied species. Consequently, the putative functions and regulatory mechanism of SBP genes in *N. cadamba* can be inferred by comparing them with their orthologous genes in well-characterized model plants like *A. thaliana* and *P. trichocarpa*. In this study, the synteny analysis of the duplicated blocks between the *N. cadamba* genome and *A. thaliana* genome indicated that 22 pairs of SBP genes were located in syntenic genomic regions, containing 13 *AtSBP* genes and 17 *NcSBP* genes (Figure 4B). To date, the majority of *AtSBP* genes, including *AtSPL2*, *AtSPL3*, *AtSPL4, AtSPL5, AtSPL7, AtSPL8, AtSPL9, AtSPL10, AtSPL11, AtSPL12, AtSPL13, AtSPL14,* and *AtSPL15,* have been well functionally characterized [15,46,47,48,49,50]. Therefore, the possible functions of the *NcSBP* homologs can be deduced from their analogous proteins, and further experimental studies are necessary to confirm these predictions.

The SBP family serve as crucial regulators of various biological and physiological processes in plants. Expression pattern analysis provides important clues for exploring the function of SBP genes in non-model plants. *NcSBP* genes exhibited wide expression across various tissues we tested, with *NcSBP1*, *NcSBP3*, and *NcSBP14* demonstrating high expression levels in most tissues (Figure 6), implying their critical importance. Accumulating evidence indicates that SBP genes are involved in the regulation of fruit development. For instance, SPL genes are expressed at higher levels in flower buds and young fruits in *Prunus mume* [51]. *SlSPL-CNR* in tomato is predominantly expressed during the ripening of fruits and plays a crucial role in promoting fruit ripening and regulating cell death [52]. In our study, *NcSBP6* exhibited extremely high expression in the fruit of *N. cadamba*, whereas the low transcript levels in other *NcSBPs* (Figure 6A,D) imply a potential regulatory role in fruit development. Given the importance of wood formation in perennial trees, we concentrated our analysis on the expression patterns observed in the xylem, cambium, and phloem during different developmental stages. Some *NcSBP* genes have high expression levels with high overlapping. For example, *NcSBP17* displayed high expression in all vascular cells at the three various stages, except in cambium at the transition stage (Figure 6B), indicating its significant role in regulating vascular cell development. *TaSPL14* from bread wheat has been identified as a crucial regulator in various vascular cell types in root, including protoxylem, protophloem, and companion cells [53]. However, information about the regulation of SBP genes in vascular tissues, especially in woody plants, is still largely unknown. Further studies are needed to investigate the specific functions of *NcSBP* on the wood formation of *N. cadamba*.

SBP genes have been characterized to play diverse, significant roles in response to various hormones and stresses. The expression of SBP genes was affected by different hormone and stress treatments. The expression of *AtSPL9* decreased under NaCl and drought treatment, and increased following recovery from these stress conditions [54], similarly to *TaSPL6* from wheat under high temperature, dehydration, and ABA stress [55]. Promoters of most *NcSBP* genes contain abundant response elements associated with hormones and stress (Figure 5B), suggesting their potential involvement in regulatory processes mediated by hormones and stress. *NcSBP14/19/20/25* were significantly increased under IAA treatment, consistent with the fact that they contained abundant auxin response element (AuxRR). Likewise, *NcSBP19/20/24* have a drought-responsive MYB binding site (MBS) element, and they were found to be up-regulated under PEG stress conditions. These results revealed consistency between the *cis*-acting regulatory elements and the expression patterns in response to hormone or stress treatments. Previous studies have revealed that SBPs are important regulators in linking hormone signaling in response to environmental stresses. *VvSBP8/13* from grape, targeted by miR156, function downstream of the ABA signaling pathway to modulate anthocyanin biosynthesis in grapevine fruit during drought conditions [56]. *CaSBP13* acts as a negative regulator of drought tolerance in pepper (*Capsicum annuum*), likely through the modulation of ROS and ABA signaling pathways [57]. In our study, certain *NcSBP* genes were activated by both hormonal signals and abiotic stressors. Specifically, *NcSBP20* and *NcSBP25* were responsive to IAA and MeJA, as well as to drought and low-temperature stress. The expression of *NcSBP10* was elevated in response to IAA, MeJA, PEG, and NaCl treatments (Figure 7 and Figure 8). In apple, many *MdSBP* genes exhibited either up-regulation or down-regulation in response to various plant hormones, including ethylene, salicylic acid (SA), MeJA, ABA, and GA [8]. In contrast, several SBP genes from tea plant, including *CsSBP5*, *CsSBP15*, *CsSBP16*, and *CsSBP19*, were repressed under cold, drought, ABA, GA, and MeJA treatments [43]. These results indicate that the SBP gene family may play a significant role in the interplay between various plant hormones and environmental stresses.

## 5. Conclusions

In summary, a total of 27 *NcSBP* genes were identified in *N. cadamba* and classified into eight clades according to phylogenetic analysis. Their gene structures, conserved motifs, and collinearity were also investigated. The expression profile of *NcSBPs* across various tissues indicated their potential roles in the growth and development of *N. cadamba*. Furthermore, the analysis of *cis*-acting elements and expression patterns of *NcSBP* genes highlighted their significant involvement in regulating responses to different hormones and abiotic stresses. Our study lays a robust foundation for further investigation into the SBP-mediated molecular mechanisms underlying physiological developmental processes and stress responses.

## Figures and Tables

**Table 1:** Physicochemical properties of SQUAMOSA promoter-binding proteins (SBPs) in *N. cadamba*.

Gene Name	Number of Amino Acids	Molecular Weight (Da)	Theoretical Isoelectric Point (pI)	Instability Index	Aliphatic Index	Grand Average of Hydropathicity
NcSBP1	160	18,390.48	8.99	74.72	42.12	−1.209
NcSBP2	374	41,811.45	8.62	64.18	63.16	−0.686
NcSBP3	338	35,693.41	9.17	53.40	54.05	−0.661
NcSBP4	366	39,024.07	9.19	55.27	52.27	−0.716
NcSBP5	365	40,141.66	7.16	64.17	64.16	−0.626
NcSBP6	227	25,268.04	6.48	35.66	52.47	−0.681
NcSBP7	467	50,966.04	8.66	52.99	68.37	−0.479
NcSBP8	207	23,523.51	8.47	70.94	66.91	−0.597
NcSBP9	148	16,714.34	9.47	75.61	42.97	−1.120
NcSBP10	437	47,421.41	7.96	56.03	59.89	−0.635
NcSBP11	520	57,913.78	6.86	51.09	72.02	−0.592
NcSBP12	458	504,469.66	8.92	50.94	63.67	−0.599
NcSBP13	279	31,079.96	9.76	57.12	67.78	−0.617
NcSBP14	205	22,683.08	9.24	48.41	42.39	−1.141
NcSBP15	200	23,186.48	5.64	97.12	40.0	−1.400
NcSBP16	807	89,792.49	6.55	52.81	77.41	−0.399
NcSBP17	1007	112,571.72	7.72	53.35	82.6	−0.440
NcSBP18	184	20,945.47	9.07	45.69	52.01	−1.048
NcSBP19	1038	114,331.61	6.09	49.96	85.13	−0.318
NcSBP20	259	29,184.32	9.59	67.50	38.46	−1.140
NcSBP21	1007	112,572.40	7.18	55.98	80.63	−0.440
NcSBP22	324	36,282.26	6.70	62.34	60.43	−0.694
NcSBP23	464	50,676.55	8.81	52.36	60.75	−0.630
NcSBP24	464	51,786.02	8.68	49.90	66.47	−0.642
NcSBP25	552	60,659.47	7.94	72.92	61.54	−0.808
NcSBP26	530	58,574.96	8.60	56.71	58.51	−0.677
NcSBP27	218	24,525.01	6.68	83.17	40.69	−1.258

## Data Availability

The data presented in this study are available in the Supplementary Materials.

## Supplementary Materials

The following supporting information can be downloaded at: https://www.mdpi.com/article/10.3390/genes16040460/s1, Figure S1: Alignment of SBP domains of NcSBP proteins; Table S1: The accession numbers of the SBP proteins used for ML phylogenetic tree construction from different species; Table S2: Expressions of *NcSBP* gene from transcriptomic data; Table S3: The specific primers used in the qRT-PCR.
