# Supplementary material for: Genome-Wide Identification and Expression Pattern Analysis of SBP Gene Family in Neolamarckia cadamba
Source: Genes (Basel). 2025 Apr 17;16(4):460. doi: 10.3390/genes16040460 (PMC12026679; doi:10.3390/genes16040460)
Supplement: Supplementary file 1 [file genes-16-00460-s001.zip › Figure S1.pdf]

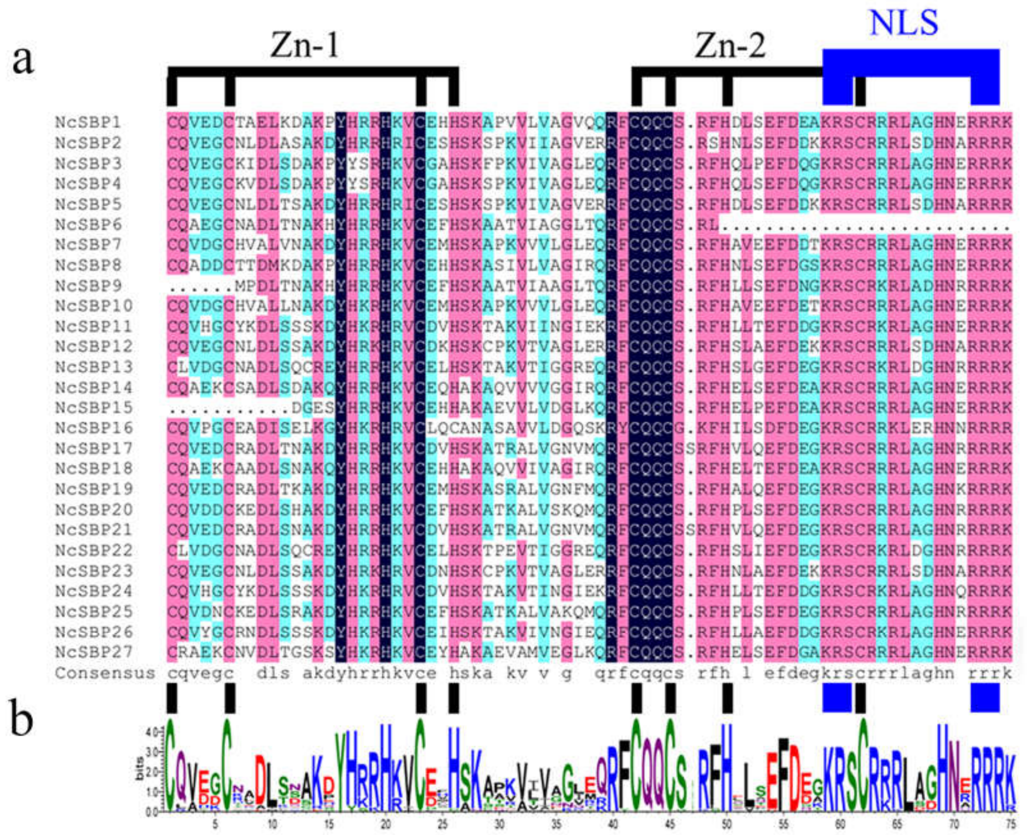

Figure S1. Alignment of SBP domains of NcSBP proteins. (a) The SBP domain sequences of NcSBP protein were aligned by DNAMAN software (version 8.0). Two conserved zinc finger structures (Zn-1, Zn-2) and nuclear localization signal (NLS) were indicated. (b) The sequence logos of the SBP domain were analyzed and visualized by Weblogo. The total height of each column of letters indicates the degree of conservation of each position, and the height of each letter indicates the relative evaluation rate of the corresponding amino acid.
