# Supplementary material for: Genome-Wide Identification and Expression Pattern Analysis of SBP Gene Family in Neolamarckia cadamba
Source: Genes (Basel). 2025 Apr 17;16(4):460. doi: 10.3390/genes16040460 (PMC12026679; doi:10.3390/genes16040460)
Supplement: Supplementary file 1 [file genes-16-00460-s001.zip › Table S1.pdf]

**Table S1 The accession numbers of the SBP proteins used for ML phylogenetic tree construction from different species**

| Species | Gene Name | Accession no. | Database |
|---|---|---|---|
| *Arabidopsis thaliana* | *AtSPL1* | At2g47070 | TAIR |
| | *AtSPL2* | At5g43270 | TAIR |
| | *AtSPL3* | At2g33810 | TAIR |
| | *AtSPL4* | At1g53160 | TAIR |
| | *AtSPL5* | At3g15270 | TAIR |
| | *AtSPL6* | At1g69170 | TAIR |
| | *AtSPL7* | At5g18830 | TAIR |
| | *AtSPL8* | At1g02065 | TAIR |
| | *AtSPL9* | At2g42200 | TAIR |
| | *AtSPL10* | At1g27370 | TAIR |
| | *AtSPL11* | At1g27360 | TAIR |
| | *AtSPL12* | At3g60030 | TAIR |
| | *AtSPL13* | At5g50570 | TAIR |
| | *AtSPL14* | At1g20980 | TAIR |
| | *AtSPL15* | At3g57920 | TAIR |
| | *AtSPL16* | At1g76580 | TAIR |
| *Populus trichocarpa* | *PtSPL1* | Potri.010G154000 | NCBI |
| | *PtSPL2* | Potri.002G002400 | NCBI |
| | *PtSPL3* | Potri.010G026200 | NCBI |
| | *PtSPL4* | Potri.008G197000 | NCBI |
| | *PtSPL5* | Potri.008G098600 | NCBI |
| | *PtSPL6* | Potri.014G114300 | NCBI |
| | *PtSPL7* | Potri.002G188700 | NCBI |
| | *PtSPL8* | Potri.002G142400 | NCBI |
| | *PtSPL9* | Potri.005G258700 | NCBI |
| | *PtSPL11* | Potri.003G172600[b] | NCBI |
| | *PtSPL12* | Potri.008G097900 | NCBI |
| | *PtSPL13* | Potri.010G154300 | NCBI |
| | *PtSPL14* | Potri.015G098900 | NCBI |
| | *PtSPL15* | Potri.012G100700 | NCBI |
| | *PtSPL16* | Potri.011G055900 | NCBI |
| | *PtSPL17* | Potri.016G048500[c] | NCBI |
| | *PtSPL18* | Potri.001G058600 | NCBI |
| | *PtSPL19* | Potri.001G055900 | NCBI |
| | *PtSPL20* | Potri.001G398200 | NCBI |
| | *PtSPL21* | Potri.002G142200 | NCBI |
| | *PtSPL22* | Potri.003G169400 | NCBI |
| | *PtSPL23* | Potri.004G046700 | NCBI |
| | *PtSPL24* | Potri.007G138800 | NCBI |
| | *PtSPL25* | Potri.011G116800[d] | NCBI |
| | *PtSPL26* | Potri.014G057700 | NCBI |
| | *PtSPL27* | Potri.014G057800 | NCBI |
| | *PtSPL28* | Potri.015G060400 | NCBI |
| | *PtSPL29* | Potri.018G149900 | NCBI |

| | | | |
|---|---|---|---|
| *Oryza sativa* | *OsSPL1* | Os01g18850 | TIGR |
| | *OsSPL2* | Os01g69830 | TIGR |
| | *OsSPL3* | Os02g04680 | TIGR |
| | *OsSPL4* | Os02g07780 | TIGR |
| | *OsSPL5* | Os02g08070 | TIGR |
| | *OsSPL6* | Os03g61760 | TIGR |
| | *OsSPL7* | Os04g46580 | TIGR |
| | *OsSPL8* | Os04g56170 | TIGR |
| | *OsSPL9* | Os05g33810 | TIGR |
| | *OsSPL10* | Os06g44860 | TIGR |
| | *OsSPL11* | Os06g45310 | TIGR |
| | *OsSPL12* | Os06g49010 | TIGR |
| | *OsSPL13* | Os07g32170 | TIGR |
| | *OsSPL14* | Os08g39890 | TIGR |
| | *OsSPL15* | Os08g40260 | TIGR |
| | *OsSPL16* | Os08g41940 | TIGR |
| | *OsSPL17* | Os09g31438 | TIGR |
| | *OsSPL18* | Os09g32944 | TIGR |
| | *OsSPL19* | Os11g30380 | TIGR |
