# Supplementary material for: Genome-Wide Identification and Expression Pattern Analysis of SBP Gene Family in Neolamarckia cadamba
Source: Genes (Basel). 2025 Apr 17;16(4):460. doi: 10.3390/genes16040460 (PMC12026679; doi:10.3390/genes16040460)
Supplement: Supplementary file 1 [file genes-16-00460-s001.zip › Table S3.pdf]

Table S3 The specific primers used in the qPCR

Gene Name	Primer sequence（5'-3'）	Amplicator length (bp)	Binding region	Efficiency (%)	Correlation Coefficient (R^2)
NcSBP3	CCTGGTGTATCTGGTGGTGAT<br>TCAAGGCCAGCAACAATGAC	227	121-347 nt	101.6	0.999
NcSBP6	TCATGTTCACCGGCGATGAA<br>AAGAAACAGTGGCCGGTTCA	102	35-135 nt	98.8	0.997
NcSBP7	AACGCCTCCACAACTCCTCGG<br>CTTCCTCTTGTCGGTCACCT	241	280-520 nt	92.3	0.996
NcSBP9	TCACAAACGCCAAGCACTAC<br>CCGGCAACTTCTTTTCCCAT	158	11-168 nt	98.4	0.993
NcSBP11	TCAGCTGTTGCCCTCAATGG<br>CCTGCTAGATGTATTCTGTG	183	1003-1185 nt	101.3	0.999
NcSBP14	GGCGGTGCAAGTTCAATGAA<br>TTGACAAAACCTTTGCCGGA	153	217-369 nt	92.4	0.993
NcSBP20	TATTCACGCAACAGCCACAACAGC<br>CATCGTGGGCGGGGAGGTT	134	122-255 nt	95.0	0.989
NcSBP22	GCCAGTTCCCATTTGCTCAA<br>TGATGAGATCCACCGCTTGT	145	806-950 nt	92.9	0.983
NcSBP25	GCCCTCTCATAATTGCAGCC<br>CCGGCTTCTTTCAGGTTGAG	130	990-1119 nt	95.5	0.984

Note: E, amplification efficiency; R^2,regression coefficient.
